# Effectiveness of NSW health get healthy telephone coaching in adults screened from general practices

**DOI:** 10.1186/s12889-024-19849-0

**Published:** 2024-09-02

**Authors:** John Attia, Natasha Weaver, Roseanne Peel, Kerry Fleming, Elizabeth Holliday, Chris Rissel, Adrian Bauman, John Wiggers, Shamasunder Acharya, Judy Luu, Penny Reeves, Mark McEvoy, Alexis Hure

**Affiliations:** 1 School of Medicine and Public Health, University of Newcastle, NSW, Australia; 2 Division of Medicine, Hunter New England Local Health District, NSW, Australia; 3 Health Research and Translation and Population Health, Hunter New England Local Health District, NSW, Australia; 4 Health Research Economics, Hunter Medical Research Institute, NSW, Australia; 5 Diabetes Service and Diabetes Alliance Hunter New England Local Health District, NSW, Australia; 6 Flinders University, Adelaide SA, and The University of Sydney, Sydney NSW, Australia; 7 School of Public Health, and the Charles Perkins Centre, University of Sydney, Sydney, 2006 Australia; 8 Department of Rural Health, LaTrobe University, Bendigo, Australia

**Keywords:** Get healthy, Weight loss, Nutrition, Physical activity, General practitioners, Pre-diabetes

## Abstract

**Background:**

The effectiveness of the NSW Health “Get Healthy Information and Coaching Service^®^^”^(Get Healthy) to facilitate weight loss on a population scale has been documented, but this was based on self-reported measures. Our study aims to test the effectiveness of the Get Healthy Service on objectively measured weight, BMI, waist circumference, and changes in other health behaviours, including nutrition, physical activity and alcohol intake.

**Methods:**

Men and women aged 40–70 years (*n* = 154) with pre-diabetes (5.7% < HbA1c < 6.5%) were referred from GP Practices to the Get Healthy Service, NSW Health. A subset (*n* = 98) participated in the “Zinc In Preventing the Progression of pre-Diabetes” (ZIPPeD) trial (ACTRN12618001120268).

**Results:**

The *self-reported* outcomes showed a statistically significant improvement from baseline to 12 months in weight (mean 2.7 kg loss, *p* < 0.001), BMI (mean 1 unit reduction, *p* < 0.001), and waist circumference (mean 4.3 cm reduction, *p* < 0.001). However, in the *objectively measured* outcomes from ZIPPeD, the differences were more modest, with point estimates of 0.8 kg mean weight loss (*p* = 0.1), 0.4 unit reduction in BMI (*p* = 0.03), and 1.8 cm reduction in waist circumference (*p* = 0.04). Bland-Altman plots indicated that discrepancies were due to a small number of participants who dramatically underestimated their weight or BMI. There were minimal changes in nutrition, physical activity, and alcohol.

**Conclusions:**

The potential benefits of Get Healthy should be interpreted with caution as we have shown significant differences between self-reported and objectively measured values. More valid and objective evidence is needed to determine the program’s effectiveness and cost-effectiveness.

**Supplementary Information:**

The online version contains supplementary material available at 10.1186/s12889-024-19849-0.

## Background

Weight loss is an important strategy for people living with overweight or obesity with, or at risk of developing, type 2 diabetes and other chronic conditions. Among these individuals, as little as 5% body weight loss can improve glycaemic control and reduce the need for diabetes medication [1]. Aimed at population health promotion, NSW Health offers the Get Healthy Information and Coaching Service (Get Healthy), supporting adults to make positive lifestyle changes and reduce excess weight, through healthy eating and physical activity.

Launched in 2009, [2] Get Healthy is a free, telephone-based service in which participants receive 10–13 individually tailored phone coaching, generally over a six-month period, delivered by university qualified health professionals, called coaches. The service was initially designed for self-referral, with a complementary clinician referral pathway subsequently added. Get Healthy covers multiple distinct programs, tailored for diabetes, pregnancy, or Aboriginal participants, which differ in content and intensity. Get Healthy is a key initiative in the State’s Healthy Eating and Living (HEAL) Strategy and the NSW Ministry of Health has invested significantly in the provision of this service [3].

There is evidence that the Get Healthy programs are effective for reducing weight and improving diet over 6 months, although these evaluations largely relied on self-reported information obtained via telephone by the coaches as part of the standard service [4]. One validation sub-study has been performed for Get Healthy, on a convenience urban sample of 38 participants. This validation compared self-reported and objectively measured height, weight, and waist circumference at six months follow-up [5]. While the study found acceptable validity for self-reported height, weight, and waist circumference, and modest reliability in the nutrition and physical activity measures, these validation results require replication.

Previously, we reported a placebo-controlled, double-blind, randomised trial testing whether zinc supplementation could improve glucose handling parameters in participants with pre-diabetes: the Zinc In Preventing the Progression of pre-Diabetes (ZIPPeD) study [6]. Although the trial did not show any effect of zinc on glucose handling, all participants (both control and intervention) were referred to the Get Healthy Service for the Type 2 Diabetes Prevention. The secondary outcomes in the ZIPPeD study (weight, BMI, and waist circumference) coincided with the outcomes targeted in Get Healthy, which provided an opportunity to independently assess the effectiveness of the Get Healthy Service for improving health markers and behaviours associated with diabetes risk, using a longitudinal cohort study design. In addition, the collection of objective measurements at participants’ General Practitioner’s (GP) office provided an opportunity to directly compare the subjectively reported and objectively measured weight, BMI, and waist circumference for the same individuals, before and after participation in Get Healthy. Self reported nutrition and physical activity data were also collected in ZIPPeD, providing the opportunity to assess the concurrent validity of these measures.

Given our randomised controlled trial (RCT) detected no effect of zinc supplementation on any outcomes, the current study combined the ZIPPeD intervention and control groups and reports the change in outcomes from baseline to 12 months for the Get Healthy measures (subjectively reported) and our ZIPPeD study measures (objectively measured). The primary aim was to test the effectiveness of the Get Healthy Service on weight, BMI, and waist circumference, and health behaviours including alcohol intake, nutrition, and physical activity. Secondly, we tested the concordance between the Get Healthy and trial data. Thirdly, we tested the effectiveness of Get Healthy on blood pressure, glycaemic control, and blood lipids at 12 months follow-up.

## Methods

### Study design and sample

The ZIPPeD randomised placebo-controlled trial (ACTRN12618001120268) was conducted within the Hunter New England Local Health District (HNELHD) from 2018 to 2021. Study recruitment was impacted by COVID-19. The ZIPPeD protocol [7] and the results [6] have been published previously. In brief, ZIPPeD included 98 participants, testing the effectiveness of 30 mg zinc gluconate daily supplementation over 12 months for improving glycaemic control in those with pre-diabetes.

Potential participants were screened by a research nurse at GP practices and those found to have pre-diabetes were offered referral to the Get Healthy Type 2 Diabetes Prevention Service (Supplementary Fig. 1). Participants who consented to Get Healthy were also invited to join the ZIPPeD trial, which included men and women aged 40–70 years with pre-diabetes and a body mass index (BMI) ≥ 28 kg/m^2^.

The Get Healthy team collected data for referred participants throughout the ZIPPeD study. These data were shared with the study team at the individual level for those who consented and participated in ZIPPeD and at the aggregate level for those who were screened and consented to Get Healthy, but not ZIPPeD. Participants who progressed to type 2 diabetes during the trial were withdrawn from the study but were encouraged to continue with Get Healthy and referred to their GP for standard care. These participants remained in the Get Healthy evaluation but did not have an objective measure of weight or other outcomes at 12 months.

This study was approved by the Hunter New England Human Research Ethics Committee, NSW, Australia (18/07/18/3.03) and registered with the University of Newcastle Human Research Ethics Committee (H-2018-0302-959).

### Data collection

The primary outcomes were weight, BMI, and waist circumference, while the secondary outcomes were serum metabolic markers (Low Density Lipoprotein (LDL), High Density Lipoprotein (HDL), Total Cholesterol, Triglycerides, Fasting Blood Glucose, Haemoglobin A1c (HBA1c) and Insulin), dietary measures, physical activity, and alcohol intake. Participants in ZIPPeD had baseline measurements performed and were followed up at 1, 6, and 12 months. Pathology request forms were mailed to participants who then presented to their nearest community pathology laboratory. At the baseline and 12-month follow-up appointments, anthropometry was collected by a trained Registered Nurse in the General Practice setting, blinded to allocation group, at the time of their appointment. Participants enrolled in Get Healthy were asked to self-report their height and weight at enrolment, and their weight at 6 and 12 month follow up, via phone calls. Instructions were to measure height without shoes, and to measure weight with only underclothes on; no other standardisation was performed.

The Dietary Questionnaire for Epidemiological Studies (DQES v2), a validated, semi-quantitative food frequency questionnaire [8] was collected at baseline and 12-month follow-up in the ZIPPeD study. The DQES v2, from the Cancer Council of Victoria is a 74-item food frequency questionnaire developed specifically for Australian adults. Data were analysed by the Cancer Council, using previously described and validated methods [8].

Physical activity data were collected in ZIPPeD using questions from the Active Australia survey, [9] including items to assess the frequency (number of sessions) and duration (minutes per week) of walking, moderate, and vigorous physical activities over the previous week. The Active Australia Survey is an established questionnaire for monitoring physical activity levels at the population level, [9] although its criterion validity against accelerometry data is limited [10, 11].

All participants enrolled in Get Healthy were asked about their alcohol consumption at baseline and completion of the program. Alcohol intake was assessed using the modified Alcohol Use Disorders Identification Test (AUDIT-C), a 3-item alcohol screen [12, 13].

### Data analysis

Characteristics of those who did and did not participate in ZIPPeD were summarised using frequency with percent (ordinal or dichotomous measures) or median with interquartile range (Q1, Q3; continuous measures) and compared using Chi-squared test or Wilcoxon/Mann-Whitney tests, respectively. The effect of Get Healthy on each outcome was estimated as the mean change from baseline to follow-up (on the scale of the link function; see below) with 95% confidence interval (CI).

Generalised linear mixed models were used for estimation, including a random intercept to account for repeated measures on participants and when appropriate, a fixed effect indicating participation in ZIPPeD (yes/no). For continuous outcomes (weight, BMI, waist circumference, and laboratory values) a normal response distribution and identity link function was used, with effects estimated as the least squares mean change from baseline to follow-up with 95% CI. For count outcomes (number of serves of fruit and vegetables, number of physical activity sessions), a Poisson response distribution with log link function was used and effect estimates were exponentiated to incidence rate ratios (IRR) with 95% CI. Regression diagnostics for the linear models included inspection of the histogram of the residuals and residual versus fitted plots. For the count outcomes, the Poisson regression assumption of equal mean and variance of the response was assessed by comparing likelihood with a negative binomial model that accommodated overdispersion of the outcome (variance > mean).

To compare Get Healthy self-reported data to ZIPPeD study measures, Spearman correlation coefficients were calculated. Systematic bias between the two methods of ascertainment was explored using Bland-Altman plots and paired t-tests. A sensitivity analysis including only completers, including those who graduated early, was also performed. Statistical programming and analyses were performed using SAS9.4 (SAS Institute, Cary, NC), with results declared statistically significant at the conventional 5% level.

Given this was a secondary analysis of a previous RCT, sample size calculations were not performed a priori. However, our total sample size (*n* = 154) should be sufficient to detect a Cohen’s d of 0.2 at 80% power and *p* = 0.05, and our ZIPPeD sample (*n* = 98) should be sufficient to detect a Cohen’s d of 0.3; both of these effects may be considered small but clinically relevant.

## Results

### Participants

**Fig. 1:**
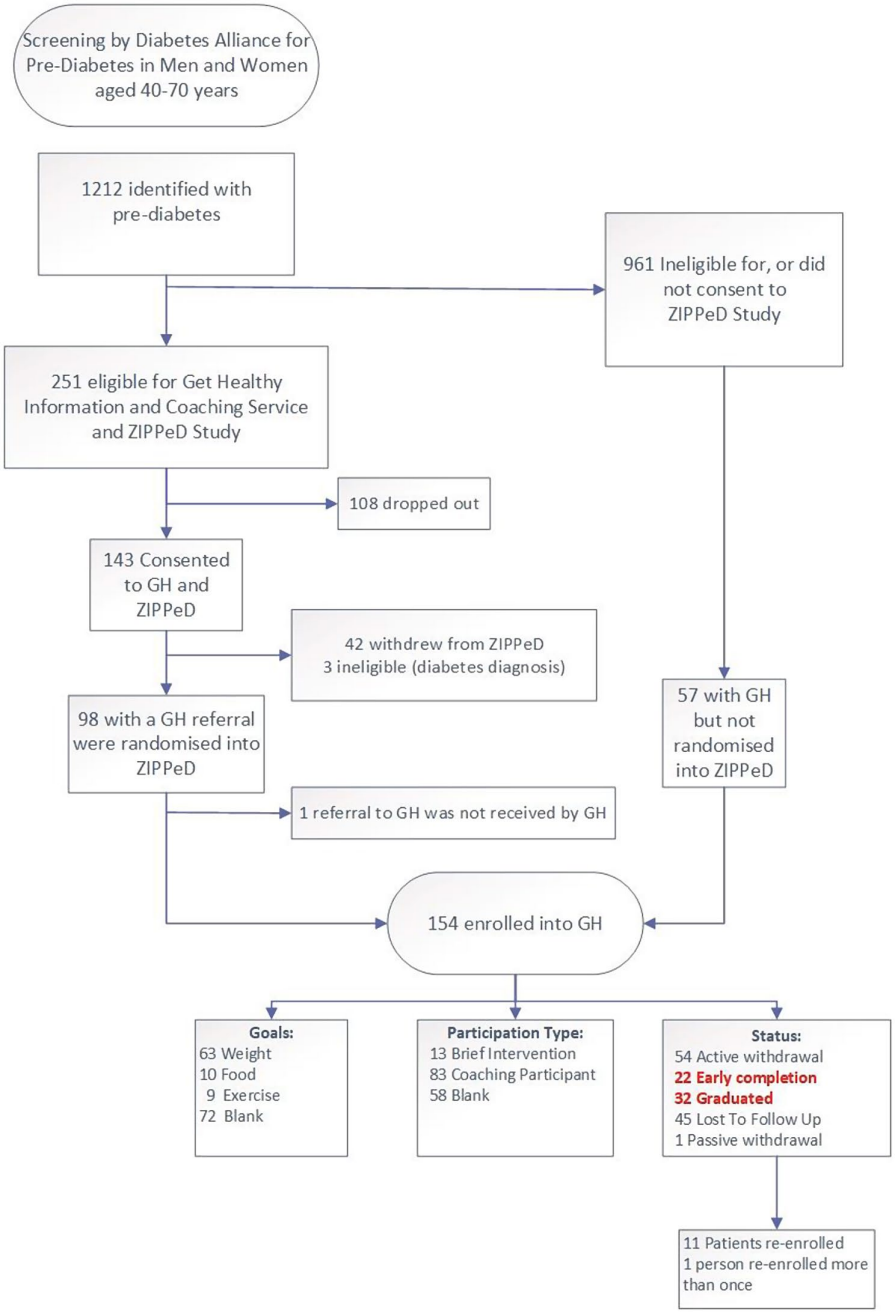
Flowchart of patient recruitment and inclusion (GH = Get Healthy)

Figure 1 describes the flow of participants who were included in this study. Overall, 1,212 people with pre-diabetes were identified through GP practices; of these 251 were eligible for the Get Healthy program and ZIPPeD trial, of whom 143 consented to ZIPPeD but only 98 completed the baseline questionnaires and were randomised. The Get Healthy referral form was lost for one ZIPPeD participant and they were not enrolled. A further 57 participants who were referred to Get Healthy but were either not eligible for ZIPPeD, or were eligible but dropped out between consent and completion of baseline questionnaires, were also included, leading to 154 participants being included in analyses.

While there were 22 early completions and 32 graduations from the program, most participants (100/154, 65%) withdrew or were lost to follow-up. On the other hand, 11 people re-enrolled in a repeat Get Healthy program.

The characteristics of those who did and did not participate in ZIPPeD were largely similar, although the trial participants tended to be younger, chose the brief intervention more often compared to the full program, and were more likely to withdraw or be lost to follow-up (Supplementary Table 1).

### Change over time in weight outcomes

Using the self-reported outcomes from Get Healthy, there was a statistically significant improvement from baseline to 12 months in weight (mean 2.7 kg loss, *p* < 0.001), BMI (mean 1 unit reduction, *p* < 0.001), and waist circumference (mean 4.3 cm reduction, *p* < 0.001) (Table 1). However, using the objectively measured outcomes from ZIPPeD the differences were more modest, with point estimates of 0.8 kg mean weight loss (*p* = 0.1), 0.4 unit reduction in BMI (*p* = 0.03), and 1.7 cm reduction in waist circumference (*p* = 0.04). Comparing the self-reported and objectively measured outcomes at baseline suggested a consistent underestimate of weight and waist circumference in self-reported data, and this discrepancy appeared to grow over time.

**Table 1:** Change over time in weight variables using self-reported (get healthy) outcomes and objectively measured (GP office) outcomes

		Baseline	Midpoint (6 months)	End (12months)	Mean change from baseline to end (95% CI)	*P*-value
**Weight variables**					Beta*	P*
Weight (kg) (mean, SD)	Self-reported (Get Healthy)	95.0 (19.0); *n* = 84	90.4 (18.9); *n* = 38	88.1 (18.3); *n* = 39	-2.7 (-3.6, -1.7)	< 0.001
	Objectively measured	95.0 (18.6); *n* = 97	na	93.1 (18.8); *n* = 72	-0.8 (-1.9, 0.2)	0.0963
Difference between self-reported and objectively measured		-0.5 (-0.9, -0.1); *p* = 0.0103		-2.4 (-3.6, -1.3); *p* = 0.0002		
BMI (kg/m^2^) (mean, SD)	Self-reported (Get Healthy)	33.4 (5.2); *n* = 84	31.2 (4.9); *n* = 38	30.7 (4.6); *n* = 39	-1.0 (-1.3, -0.6)	< 0.001
	Objectively measured	34.1 (6.3); *n* = 97	na	32.8 (6.1); *n* = 72	-0.4 (-0.9, -0.0)	0.0345
Difference between self-reported and objectively measured		-0.6 (-1.1, -0.0); *p* = 0.0404		-0.9 (-1.4, -0.5);*p* = 0.0004		
Waist circumference (cm) (mean, SD)	Self-reported (Get Healthy)	105.8 (22.1); *n* = 78	105.2 (14.0); *n* = 37	100.1 (14.2); *n* = 36	-4.3 (-6.2, -2.3)	< 0.001
	Objectively measured	110.0 (13.6); *n* = 97	na	107.7 (14.2); *n* = 72	-1.7 (-3.4, -0.1)	0.0412
Difference between self-reported and objectively measured		-4.6 (-9.1, -0.2); *p* = 0.0425		-4.1 (-7.7, -0.6); *p* = 0.0236		

*P-value from Linear Mixed Model with random intercept for participant, includes fixed effect for consent to ZIPPED

### Change over time in alcohol, nutrition, and physical activity

Repeat AUDIT scores indicated a statistically significant reduction in alcohol consumption (0.67 reduction in AUDIT score (0.50, 0.90), see Table 2). There was no improvement from baseline to 12 months in the number of serves of vegetables and fruits, using either self-reported measures or from the food frequency questionnaire (Table 2).

For physical activity, the Get Healthy measures showed an improvement in the number of self-reported episodes per week of overall physical activity and moderate to vigorous physical activity by 12 months. However, this was not concordant with the physical activity data from ZIPPeD (obtained via validated instrument), which showed no change in physical activity over time (Table 2).

**Table 2:** Change over time in nutrition and physical activity using self-reported (get healthy) outcomes and objectively measured (GP office) outcomes

		Baseline	Midpoint (6 months)	End (12 months)	Mean change from baseline to end (95% CI)	*P*-value
**Nutrition variables**					Exp(beta)**	P**
Vegetables (serves/day) (mean, SD)	Self-reported	2.7 (1.6); *n* = 54	na	3.1 (1.3); *n* = 39	1.15 (0.90, 1.50)	0.2473
	Questionnaire based	4.5 (1.3); *n* = 97	na	4.5 (1.2); *n* = 68	1.01 (0.84, 1.21)	0.9149
Fruit (serves/day) (mean, SD)	Self-reported	1.4 (1.2); *n* = 54	na	1.8 (1.0); *n* = 39	1.22 (0.87, 1.72)	0.2399
	Questionnaire based	3.2 (1.2); *n* = 97	na	3.3 (1.3); *n* = 68	1.03 (0.83, 1.27)	0.7844
AUDIT-C score	Self-reported	2.3 (2.1); *n* = 73	na	1.6 (2.1); *n* = 38	0.67 (0.50, 0.90)	0.0112
**Physical activity**					Exp(beta)**	P**
Walking (times/week)	Self-reported	1.8 (2.3); *n* = 59	na	3.0 (2.6); *n* = 38	1.40 (1.02, 1.93)	0.0377
	Questionnaire based	3.6 (3.1); *n* = 97	3.6 (2.9); *n* = 82	3.7 (3.0); *n* = 72	1.00 (0.85, 1.18)	0.9677
Mod/vigorous (times/week)	Self-reported	0.5 (1.5); *n* = 56	3.2 (3.2); *n* = 20	2.6 (3.1); *n* = 38	4.33 (2.67, 7.02)	< 0.0001
	Questionnaire based	3.5 (3.5); *n* = 97	3.8 (3.6); *n* = 82	3.9 (3.5); *n* = 72	1.01 (0.86, 1.19)	0.9118

**P-value from Generalised Linear Mixed Model with random intercept for participant, assuming Poisson distribution with log link function, fixed effect for consent to ZIPPED

### Concordance between self-report and objective measures

**Fig. 2:**
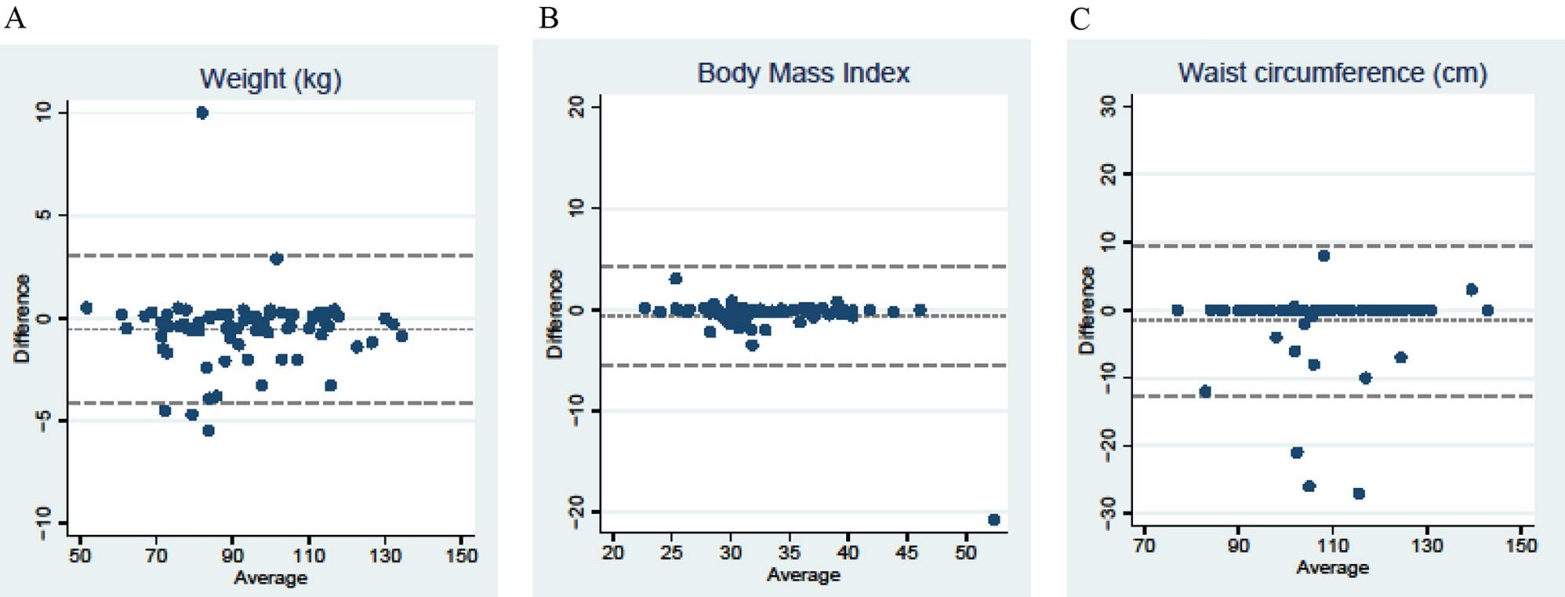
Bland-Altman plots for weight, BMI and waist circumference. **A**. Bland Altman plots for weight. **B**. Bland Altman plots for BMI. **C**. Bland Altman plots for waist circumference

We directly compared concordance between self-reported and objectively measured weight outcomes. Spearman correlation coefficients were very high at 0.98 for weight, 0.89 for BMI, and 0.92 for waist circumference. Bland-Altman plots were used to explore systematic bias in self-reported data (Fig. 2). Although most participants reported accurately, with most points falling on the line of no difference (i.e. no discrepancy between the two methods), for each measure there was a small number who had highly discordant measures of weight or waist circumference, which skewed the average difference. This led to an average underestimate of 0.5 kg weight, 0.6 units BMI, and 1.58 cm in waist circumference in self-reported data. Extreme minimum values indicate some large underestimates, including a 5.5 kg discrepancy in weight, 21-unit discrepancy in BMI, and 27 cm discrepancy in waist circumference (Table 3). All values were within the plausible range and hence remained in the analyses.

**Table 3:** Bland-Altman plots for weight outcomes. The mean differences (GetHealthy – Zipped) are all negative which indicates that, on average, participants underestimated their values in the self-reported GetHealthy variable compared to the objectively-measured zipped variable

Comparison: GH – Z	*N* pairs	Mean (SD)	Median (IQR)	Min, Max	Paired t-test^a^
Weight	78	-0.54 (1.81)	-0.40 (-0.90, 2.0)	-5.5, 10.0	*P* = 0.0103
BMI	78	-0.58 (2.45)	-0.18 (0.50, 0.04)	-20.8, 3.05	*P* = 0.0404
Waist circ	71	-1.58 (5.55)	0.0 (0.0, 0.0)	-27.0, 8.0	*P* = 0.0188

^a^ t-test is robust against the presence of a few outliers. Here, the distribution is symmetric apart from a few outliers

### Change over time in metabolic outcomes (ZIPPeD only)

There was no difference in any of the metabolic (lipids and glycaemic markers) or physical measures (blood pressure) after the Get Healthy program, except for a 3.7 mmHg reduction in mean diastolic blood pressure (Supplementary Table 2).

### Sensitivity analysis for completers only

Analyses of the subset of participants who completed the Type 2 Diabetes Prevention Program, including early completions, showed very similar results as the main analysis (see Table 4).

**Table 4:** Sensitivity analysis of change over time in weight variables using self-reported get healthy data for completers (early completion or graduated); ZIPPeD and non-ZIPPeD participants combined

	Baseline	Midpoint	End	Mean change (end v baseline) 95% CI	*P*-value
**Weight variables**				Beta*	P*
Weight (kg) (mean, SD)	89.0 (18.1); *n* = 42	88.3 (17.5); *N* = 32	86.8 (17.9); *N* = 37	-2.7 (-3.7, -1.7)	< 0.0001
BMI (m/kg2) (mean, SD)	31.2 (4.2); *N* = 42	30.6 (4.2); *N* = 32	30.2 (4.3); *N* = 37	-1.0 (-1.3, -0.6)	< 0.0001
Waist circumference (cm) (mean, SD)	100.1 (21.4); *N* = 37	103.3 (12.4); *N* = 31	98.9 (13.5); *N* = 36	-4.3 (-6.5, -2.1)	0.0002
**Nutrition variables**				Exp(beta)**	P**
Vegetables (serves/day) (mean, SD)	2.5 (1.5); *N* = 28	No data	3.2 (1.3); *N* = 37	1.29 (0.94, 1.76)	0.1115
Fruit (serves/day) (mean, SD)	1.2 (1.1); *N* = 28	No data	1.8 (1.0); *N* = 28	1.53 (0.99, 2.37)	0.0561
AUDIT-C score (mean, SD)	2.3 (2.0); *N* = 38	No data	1.6 (2.1); *N* = 38	0.64 (0.45, 0.90)	0.0118
**Physical activity**				Exp(beta)**	P**
Walking (times/week) (mean, SD)	1.9 (2.7); *N* = 29	No data	3.0 (2.7); *N* = 36	1.37 (0.96, 1.96)	0.0803
Moderate/Vigorous (times/week) (mean, SD)	0.6 (2.0); *N* = 28	3.2 (3.2); *N* = 20	2.6 (3.1); *N* = 36	4.70 (2.71, 8.16)	< 0.0001

*P-value from Linear Mixed Model with random intercept for participant, includes fixed effect for consent to ZIPPED

**P-value from Generalised Linear Mixed Model with random intercept for participant, assuming Poisson distribution with log link function, fixed effect for consent to ZIPPED

## Discussion

The findings of this study suggest the participants who engaged with and completed the Get Healthy Type 2 Diabetes Prevention Program showed small reductions in weight, waist circumference and alcohol intake and increases in moderate to physical activity at the 12 month follow up. However, the 65% drop-out rate and loss to follow-up for the cohort referred to Get Healthy was high, albeit consistent with other weight loss interventions [14]. Self-reported improvements in weight, BMI, and waist circumference in the Get Healthy Type 2 Diabetes Prevention Program were very similar to previous reports [4, 15] but larger than those obtained when these parameters were objectively measured. It is important to note that some of this study occurred during the worst of the COVID-19 lockdowns in NSW. Weight maintenance during this time may actually represent a win given data suggest that weight tended to increase more generally in the Australian population [16]. However, potential to either gain or lose weight during COVID isolation, depending on context, has also been described [17].

The potential benefits of Get Healthy should be interpreted with caution as we have shown differences stemming from some participants underestimating their weight and waist circumference, with the magnitude of underreporting growing over time. When measured objectively in ZIPPeD, weight loss did not reach statistical significance; a post-hoc sample size calculation indicates that we had 80% power to detect a Cohen’s d of 0.3, so a difference smaller than this may have been missed. Underreporting weight and overreporting height has been observed previously in other studies compared to objective measures [18, 19]. Yoong et al. have reported on discrepancies between self-report and objective measurements, ascribing it to social desirability bias [20]. Others have found that the underestimation occurs more with those at higher BMI [21]. People generally wish to appear to be adhering to the advice and it is possible that the rapport that developed with Get Healthy coaches during follow-up accentuated this tendency. The small proportion of extreme values was sufficient to skew the average and, at best, overstate the effect of Get Healthy, or at worst, create a positive effect where there was none. It is therefore difficult to determine whether the apparent effectiveness of Get Healthy Type 2 Diabetes Prevention Program was completely negated by this self-report bias, or whether it was reduced in magnitude but still important at a population level. Larger objective validation of the GHS over longer time frames is required. The pattern of underreporting is worth noting. For example, the 3 individuals with the greatest degree of underreporting weight are all on the lower range of weight (70–90 kg), and have virtually no difference in reported vs. measured BMI, while the one with the greatest discrepancy in BMI (22 units) accurately reported weight. The 3 with the greatest discrepancy (underreporting) in waist circumference were all in the middle of the range, and showed virtually no discrepancy in weight. This pattern suggests that individuals are not consistently misrepresenting their values, e.g. social desirability bias, but rather that the discrepancies are more likely due to error. However, given the spread of discrepancies is not even, i.e. more likely to underreport than overreport compared to measured values, this suggests a mild degree of bias.

While it is possible that this discrepancy between self-report and objectively measured values occurred only in the subgroup enrolled in ZIPPeD due to selection bias, and would not be seen in those normally enrolling in Get Healthy, this seems unlikely for 2 reasons: (a) the characteristics of those who participated in ZIPPeD were largely similar to those who participated in Get Healthy only (with some minor differences, see Supplementary Table 1); and (b) those participating in a trial would be expected to be more motivated, health conscious and compliant.

It is interesting to note that for both the nutrition and physical activity outcomes, the ZIPPeD measures were consistently higher than the self-reported Get Healthy values. Given that neither set of values was objectively measured it is difficult to know which is the more valid estimate. We saw a similar pattern of self-reporting in physical activity, with people overstating the number of episodes per week and even more so for the number of episodes of moderate to vigorous activity.

Given the tendency to favourably report outcomes, it is also important to question whether a similar pattern may be seen with alcohol. Get Healthy has previously been modified to target alcohol consumption, [22] and AUDIT scores in this study did show a significant improvement over time. Because the AUDIT depends on self-report, it will be important to assess whether this effect was again driven by social desirability bias and bias in self-report, or whether objective measures confirm this apparent beneficial effect.

The high rate of drop-out (65%) is not encouraging but could have been related to COVID-19. Nevertheless, it would be important to explore other ways to make the program more engaging, perhaps through principles of gamification, having an accompanying app, or via partnering with others who are doing the program to add a social dimension [23].

The statistically significant findings in this study should be interpreted cautiously in the context of multiple comparisons and high rates of missing data. While this evaluation uses pre and post data from a relatively small cohort referred to the Get Healthy Type 2 Diabetes Prevention Program, it represents a larger sample than the previous Get Healthy validation study (for Standard Coaching) [5] and there have been no RCTs or evaluations of the Get Healthy Type 2 Diabetes Prevention Program, [24] although a combination of study designs may be necessary to determine real-world effectiveness of this scaled intervention [25]. Given the likelihood that missing data may be in those who are least likely to participate in the program, it is worth noting that the direction of bias is towards the null, i.e. the discrepancy between self-reported and objectively measured values may be even higher than stated here.

Given the significant investment in operating the Get Healthy service, it is important that further, high quality evidence be generated about its effectiveness. The service is well established, has good reach with culturally and linguistically diverse, and disadvantaged populations, [26, 27] has been adapted for Indigenous populations, [28, 29] and can be easily integrated into GP practice [30]. More valid and objective evidence is needed to determine the program’s effectiveness.

### Electronic supplementary material

Below is the link to the electronic supplementary material.

Supplementary Material 1

Supplementary Material 2

Supplementary Material 3

## Data Availability

The datasets used and/or analysed during the current study are available from the corresponding author on reasonable request.

## References

[1] Klein S, Sheard NF, Pi-Sunyer X, Daly A, Wylie-Rosett J, Kulkarni K, Clark NG. Weight Management through Lifestyle Modification for the Prevention and Management of type 2 diabetes: rationale and strategies: a statement of the American Diabetes Association, the North American Association for the Study of Obesity, and the American Society for Clinical Nutrition. Diabetes Care. 2004;27(8):2067–73. 15277443 10.2337/diacare.27.8.2067

[2] O’Hara BJ, Bauman AE, King EL, Phongsavan P. Process evaluation of the advertising campaign for the NSW Get Healthy Information and Coaching Service. Health Promot J Austr. 2011;22(1):68–71. 21717841 10.1071/HE11068

[3] NSW Government. A helping hand to get healthy in 2019. In: NSW Health, editor.; 2019.

[4] O’Hara BJ, Phongsavan P, Eakin EG, Develin E, Smith J, Greenaway M, Bauman AE. Effectiveness of Australia’s get healthy information and Coaching Service: maintenance of self-reported anthropometric and behavioural changes after program completion. BMC Public Health. 2013;13:175. 23442338 10.1186/1471-2458-13-175 PMC3598693

[5] O’Hara BJ, Phongsavan P, Venugopal K, Eakin EG, Eggins D, Caterson H, King L, Allman-Farinelli M, Haas M, Bauman AE. Effectiveness of Australia’s get healthy information and Coaching Service(R): translational research with population wide impact. Prev Med. 2012;55(4):292–8. 22885323 10.1016/j.ypmed.2012.07.022

[6] Attia JR, Holliday E, Weaver N, Peel R, Fleming KC, Hure A, Wiggers J, McEvoy M, Searles A, Reeves P, Ranasinghe P, Jayawardena R, Samman S, Luu J, Rissel C, Acharya S. The effect of zinc supplementation on glucose homeostasis: a randomised double-blind placebo-controlled trial. Acta Diabetol. 2022;59(7):965–75. 35451678 10.1007/s00592-022-01888-x PMC9026040

[7] Peel R, Hure A, Wiggers J, McEvoy M, Holliday E, Searles A, Reeves P, Ranasinghe P, Jayawardena R, Samman S, Acharya S, Luu J, Rissel C, Attia J. Zinc in preventing the progression of pre-diabetes (ZIPPeD Study) - study protocol for a randomised placebo-controlled trial in Australia. Trials. 2019;20(1):219. 30992081 10.1186/s13063-019-3317-4 PMC6466783

[8] Giles G, Ireland P. Dietary Questionnaire for Epidemiological Studies (Version 2) The Cancer Council Victoria. Melbourne, Australia. 1996.

[9] Australian Institute of Health and Welfare. The active Australia survey: a guide and manual for implementation, analysis and reporting. Australian Institute of Health and Welfare; 2003.

[10] Curtis RG, Olds T, Plotnikoff R, Vandelanotte C, Edney S, Ryan J, Maher C. Validity and bias on the online active Australia survey: activity level and participant factors associated with self-report bias. BMC Med Res Methodol. 2020;20(1):6. 31924171 10.1186/s12874-020-0896-4 PMC6954551

[11] Vandelanotte C, Duncan MJ, Stanton R, Rosenkranz RR, Caperchione CM, Rebar AL, Savage TN, Mummery WK, Kolt GS. Validity and responsiveness to change of the active Australia Survey according to gender, age, BMI, education, and physical activity level and awareness. BMC Public Health. 2019;19(1):407. 30991980 10.1186/s12889-019-6717-1 PMC6466730

[12] NICE Public Health Guidance 24. Alcohol use disorders: preventing the development of hazardous and harmful drinking: NICE. London: National Institute for Health and Clinical Excellence; 2010. https://www.nice.org.uk/guidance/ph24.

[13] Bush K, Kivlahan DR, McDonell MB, Fihn SD, Bradley KA. The AUDIT alcohol consumption questions (AUDIT-C): an effective brief screening test for problem drinking. Ambulatory Care Quality Improvement Project (ACQUIP). Alcohol Use disorders Identification Test. Arch Intern Med. 1998;158(16):1789–95. 9738608 10.1001/archinte.158.16.1789

[14] Moroshko I, Brennan L, O’Brien P. Predictors of dropout in weight loss interventions: a systematic review of the literature. Obes Rev. 2011;12(11):912–34. 21815990 10.1111/j.1467-789X.2011.00915.x

[15] O’Hara BJ, Phongsavan P, McGill B, Maxwell M, Ahmed N, Raheb S, Bauman AE. The NSW Get Healthy Information and Coaching Service: the first five years. NSW Ministry of Health & Prevention Research Collaboration: University of Sydney 2014.

[16] IPSOS. More than a third of Australians have gained weight during the pandemic – Ipsos survey. 2021.

[17] Kuk JL, Christensen RAG, Kamran Samani E, Wharton S. Predictors of weight loss and weight gain in Weight Management patients during the COVID-19 pandemic. J Obes. 2021;2021:4881430. 34956673 10.1155/2021/4881430 PMC8709769

[18] Fry JM, Temple JB. Discrepancies in self-reported and measured anthropometric measurements and indices among older australians: prevalence and correlates. BMC Public Health. 2022;22(1):1928. 36253740 10.1186/s12889-022-14326-y PMC9575622

[19] Australian Bureau of Statistics. Self-reported height and weight [Internet]. 2018.

[20] Yoong SL, Carey ML, D’Este C, Sanson-Fisher RW. Agreement between self-reported and measured weight and height collected in general practice patients: a prospective study. BMC Med Res Methodol. 2013;13(1):1–8. 23510189 10.1186/1471-2288-13-38 PMC3599990

[21] Ng SP, Korda R, Clements M, Latz I, Bauman A, Bambrick H, Liu B, Rogers K, Herbert N, Banks E. Validity of self-reported height and weight and derived body mass index in middle-aged and elderly individuals in Australia. Aust N Z J Public Health. 2011;35(6):557–63. 22151163 10.1111/j.1753-6405.2011.00742.x

[22] Moore R, Ahmed N, Russell L, Rissel C. Developing a new get Healthy Service program on reducing risky alcohol consumption. Public Health Res Pract. 2016;26(4). 10.17061/phrp2641647 27714390

[23] Jain B, Bajaj SS, Stanford FC. Randomized clinical trials of weight loss: pragmatic and digital strategies and innovations. Contemp Clin Trials. 2022;114:106687. 35085830 10.1016/j.cct.2022.106687 PMC8785263

[24] NSW Health. Get Healthy Publications and Research. 2023. https://www.gethealthynsw.com.au/health-professionals/publications-and-research/

[25] Kennedy-Martin T, Curtis S, Faries D, Robinson S, Johnston J. A literature review on the representativeness of randomized controlled trial samples and implications for the external validity of trial results. Trials. 2015;16:495. 26530985 10.1186/s13063-015-1023-4 PMC4632358

[26] O’Hara BJ, Phongsavan P, Venugopal K, Bauman AE. Characteristics of participants in Australia’s get healthy telephone-based lifestyle information and coaching service: reaching disadvantaged communities and those most at need. Health Educ Res. 2011;26(6):1097–106. 21987479 10.1093/her/cyr091

[27] O’Hara BJ, Eggins D, Phongsavan P, Milat AJ, Bauman AE, Wiggers J. Piloting proactive marketing to recruit disadvantaged adults to a community-wide obesity prevention program. Public Health Res Pract. 2015;25(2):e2521521. 25848739 10.17061/phrp2521521

[28] Quinn E, O’Hara BJ, Ahmed N, Winch S, McGill B, Banovic D, Maxwell M, Rissel C. Enhancing the get healthy information and coaching service for Aboriginal adults: evaluation of the process and impact of the program. Int J Equity Health. 2017;16(1):168. 28877697 10.1186/s12939-017-0641-8 PMC5586001

[29] Winch S, Ahmed N, Rissel C, Maxwell M, Coutts J, Lucas K. The reach and flow of health information in two Aboriginal communities: a social network analysis. Aust J Prim Health. 2017;23(2):189–95. 27756447 10.1071/PY16024

[30] O’Hara BJ, Phongsavan P, Rissel C, Hardy LL, Zander A, Greenaway M, Bauman AE. Role of general practice in the utilisation of the NSW Get Healthy Information and Coaching Service. Aust J Prim Health. 2015;21(2):182–8. 24456670 10.1071/PY13154

