# Supplementary material for: Effectiveness of NSW health get healthy telephone coaching in adults screened from general practices
Source: BMC Public Health. 2024 Sep 2;24:2372. doi: 10.1186/s12889-024-19849-0 (PMC11368026; doi:10.1186/s12889-024-19849-0)
Supplement: Supplementary file 1 — Supplementary Material 1 [file 12889_2024_19849_MOESM1_ESM.pdf]

get healthy®
Information & Coaching Service

MAKE HEALTHY NORMAL

NSW GOVERNMENT

ZIPPeD STUDY REFERRAL

RETURN THE COMPLETED FORM TO:
Fax: 1300 013 242
or Email: contact@gethealthynsw.com.au

Simply call 1300 806 258
www.gethealthynsw.com.au

Disclaimer: By completing this form you consent to this information being sent to the Get Healthy Information and Coaching Service®, and consent for the Service staff to contact you.

Referrer Details (print or stamp below)

Name:

Profession:

Organisation/Hospital:

Address (for feedback letters):

Postcode:

Phone Number:

Email:

Patient details

Please print or affix patient sticker on top

First Name:

Surname:

DOB:

Gender: ☐ Female ☐ Male

Address:

Suburb:

Postcode:

Tel. home:

Tel. mobile:

Email:

Are you of Aboriginal or Torres Strait Islander origin?

☐ No

☐ Yes, Aboriginal

☐ Yes, Torres Strait Islander

☐ Yes, both Aboriginal and Torres Strait Islander

Preferred goal

☐ Physical Activity ☐ Weight Management

☐ Healthy Eating ☐ Alcohol Reduction

Is an interpreter required?

☐ No ☐ Yes

Specify language:

When is the best time to call?

☐ am ☐ pm

Are you pregnant? ☐ No ☐ Yes

General comments

Please describe any health condition(s)/ impairment(s) which may affect what the patient eats or how physically active they can be:

Current body measurements (Optional)

Waist circumference (cm):

Height (cm):

Weight (kg):

If pregnant:

Pre-pregnancy weight (kg):

Gestational Age (wks):

Feedback letters (optional)

☐ I, the health professional named above, would like feedback letters on the above patient's contact with the Service.

Date:
