# Supplementary material for: Effectiveness of NSW health get healthy telephone coaching in adults screened from general practices
Source: BMC Public Health. 2024 Sep 2;24:2372. doi: 10.1186/s12889-024-19849-0 (PMC11368026; doi:10.1186/s12889-024-19849-0)
Supplement: Supplementary file 2 — Supplementary Material 2 [file 12889_2024_19849_MOESM2_ESM.pdf]

Supplementary Table 1. Characteristics of Get Healthy participants who did and did not participate in ZIPPeD

Variable	Level or statistic	Consented to ZIPPED (n=97)	Did not consent to ZIPPED (n=57)	P-value*
**Socio-demographics**				
Regionality	Major cities	78 (80%)	50 (89%)	0.179
	Inner Regional	20 (20%)	6 (11%)	
	Missing	1	1	
Age	Median (Q1,Q3)	63.0 (56.6, 67.0)	58.8 (50.2, 65.1)	
	Missing	0	0	
Gender	Females	54 (56%)	30 (55%)	1.000
	Males	43 (44%)	25 (45%)	
	Missing	0	2	
Indigenous status	Aboriginal	1 (1.2%)	2 (7.1%)	0.154
	No	83 (99%)	26 (93%)	
	Missing	13	29	
Language	English	82 (98%)	16 (89%)	0.142
	Other	2 (2.4%)	2 (11%)	
	Missing	13	39	
Education	University Bachelor or above (AQF 7-10)	11 (15%)	0	1.000
	Certificate or Diploma (AQF 1-6)	26 (37%)	1 (33%)	
	Year 12 or less	34 (48%)	2 (66%)	
	Missing	26	54	
Employment	Full/part-time	26 (34%)	1 (20%)	0.024
	Unemployed	9 (12%)	0	
	Retired	35 (46%)	1 (20%)	
	Other	6 (7.9%)	3 (60%)	
	Missing	21	52	
AUSDRisk score	Median (Q1, Q3)	23 (18, 26)	24 (13, 27)	
	Missing	0	51	
**Program**				
Participant type	Brief intervention	6 (7.3%)	7 (50%)	<.001
	Coaching	76 (93%)	7 (50%)	
	Missing	15	43	
Goal	Physcial activity-related	9 (12%)	0	N/A
	Nutrition-related	10 (13%)	0	
	Weight-related	57 (75%)	6 (100%)	
	Missing	21	51	
Status	Early completion	20 (21%)	2 (3.5%)	<.001
	Graduation	25 (26%)	7 (20%)	
	Withdrawal	42 (43%)	12 (21%)	
	LTFU	10 (10%)	35 (61%)	
	Terminated	0	1 (1.8%)	
