# Supplementary material for: Effectiveness of NSW health get healthy telephone coaching in adults screened from general practices
Source: BMC Public Health. 2024 Sep 2;24:2372. doi: 10.1186/s12889-024-19849-0 (PMC11368026; doi:10.1186/s12889-024-19849-0)
Supplement: Supplementary file 3 — Supplementary Material 3 [file 12889_2024_19849_MOESM3_ESM.pdf]

Supplementary Table 2. ZIPPeD blood tests and physical measure: change over time for the intervention and control groups combined

	Baseline (n=97)	1 month (n=94)	6 months (n=82)	12 months (n=72)	Mean change 12m vs baseline (95% CI)	P-value*
**Physical & pathology measures**					Beta	P*
SBP	133.9 (16.6)	n/a	n/a	132.7 (14.3)	-1.2 (-5.4, 3.0)	0.5658
DBP	82.8 (9.3)	n/a	n/a	78.7 (8.6)	-3.7 (-5.8, -1.5)	0.0011
LDL	3.0 (0.9)	3.0 (0.9)	3.1 (1.1)	2.9 (1.1)	-0.06 (-0.20, 0.08)	0.4108
HDL	1.3 (0.4)	1.3 (0.3)	1.4 (0.6)	1.3 (0.3)	-0.01 (-0.10, 0.07)	0.7524
Total cholesterol	5.1 (1.1)	5.1 (1.1)	5.1 (1.2)	5.0 (1.2)	-0.11 (-0.27, 0.06)	0.1997
Triglycerides	1.8 (0.9)	1.7 (0.7)	1.7 (0.8)	1.7 (0.8)	-0.08 (-0.23, 0.06)	0.2634
FBG	5.9 (0.8)	5.7 (0.6)	5.7 (0.7)	5.8 (0.7)	-0.04 (-0.18, 0.10)	0.5925
Hba1c	5.9 (0.2)	5.8 (0.3)	5.8 (0.3)	5.9 (0.3)	0.04 (-0.02, 0.10)	0.1681
Insulin	13.7 (7.0)	14.0 (7.3)	13.9 (7.4)	13.5 (7.0)	0.12 (-0.89, 1.14)	0.8109
**HOMA2 parameters**					Beta	P*
%b	103.0 (36.1)	110.2 (37.5)	108.9 (39.1)	104.0 (33.5)	1.24 (-4.61, 7.10)	0.6759
%s	72.7 (44.4)	70.3 (40.7)	72.6 (44.7)	70.5 (34.9)	-1.80 (-7.82, 4.22)	0.5572
ir	1.8 (0.9)	1.9 (0.9)	1.8 (1.0)	1.8 (0.9)	0.01 (-0.12, 0.15)	0.8514
